# The impact of death priming on climate change denial: a preliminary investigation

**DOI:** 10.12688/f1000research.151742.1

**Published:** 2024-07-03

**Authors:** Zach Gerber, David Anaki

**Affiliations:** 1 Psychology, Bar Ilan University, Ramat-Gan, 52900, Israel; 2 The Leslie and Susan Gonda (Goldschmied) Multidisciplinary Brain Research Center, Bar-Ilan University, Ramat-Gan, 52900, Israel

**Keywords:** Climate Change Denial, Terror Management Theory, Political Orientation.

## Abstract

**Background:**

Climate change denial (CCD) has been found to be more pronounced among individuals with a conservative political orientation. Terror Management Theory posits that an individual’s worldview serves as a primary defense mechanism in coping with existential threats, such as the reality of climate change. Drawing on these premises, we conducted preliminary research investigating the impact of death priming on CCD from the Terror management theory perspective.

**Methods and results:**

We administered a death priming task and measured CCD in its immediate wake or following a delay task. We confirmed among 219 Amazon’s MTurk participants that immediately following death priming, CCD was reduced among all participants. In the delayed death priming condition, we acquired preliminary evidence that CCD was heightened among Republican participants.

**Conclusions:**

These findings demonstrate the relevance of death priming to CCD tendencies and potentially spawn future research regarding CCD as a particular form of coping with existential threats.

We live in a geological epoch in which human corporal existence has become prominent in impacting the Earth’s environment and climate ( Lewis & Maslin, 2015). Climate science broadly indicates that in our technological quest for dominance over Earth’s natural resources, we have pushed our way of life beyond the brink of sustainability to the point of irretrievable loss ( Moser, 2020). However, despite hard evidence underscoring the climate crisis, denial and rejection of the scientific knowledge that points to its dangers are prevalent among individuals ( Jylhä et al., 2022). This phenomenon, known as climate change denial (CCD), has received growing attention in psychological science and discourse ( Wullenkord, 2022).

CCD has been studied with reference to ideological variables and sociopolitical identities: Higher and more robust scores on different climate change denial measures have been typically found among conservatives and right-wing adherents and activists ( Jylhä et al., 2016; Clarke et al., 2019; Wullenkord, 2022). This is presumably because climate mitigation solutions challenge central conservative ideals such as a free-market philosophy and personal freedoms, thereby threatening central aspects to their way of life ( Norgaard, 2019). In addition, CCD has been considered a coping mechanism with the reality of climate change which augments individuals’ sense of personal mortality and existential peril ( Wolfe & Tubi, 2019; Gerber, 2023). Therefore, in the current study, we explored how CCD converges with Terror Management Theory (TMT, Greenberg et al., 1986), a leading paradigm in psychological science regarding mortality, existential threat ( Schindler et al., 2023), and in particular climate related behaviors ( Smith et al. 2022).

TMT accounts for the unique and paradoxical cognitive predicament that human beings undergo. We share the relentless striving for survival with all living things, yet solely experience the capacity for self-awareness regarding our mortality and the inevitability of death. Acknowledging the ultimate predetermined failure of all striving for survival creates paralyzing anxiety. Research indicates that individuals utilize defense mechanisms to push thoughts of death out of their conscious awareness ( Pyszczynski et al., 2015). Specifically, TMT researchers predict that when reminded of mortality, people will seek out one of two defense modes that comprise the dual defense model. The first is proximal defenses, which reflect logical steps to reduce mortality as an imminent threat. For example, in the wake of immediate death priming, participants indicated their heightened intention to utilize sun lotion during sun exposure to ensure a longer life ( Routledge et al., 2004). The second approach, distal defenses, reflects a subconscious awareness of mortality and the anxiety stemming from the ultimate inevitability of death. These defenses are expressed by imbuing one’s way of life or body with aesthetic value or meaning, fulfilling the promise of at least symbolic immortality. Importantly, proximal defenses emerge shortly after reminders of death occur, while distal defenses transpire in response to death reminders only after a delay and distraction. Indeed, in the wake of death priming and a delay task, participants indicated their reduced intention to use sun lotions when exposing their bodies to the sun.

TMT research has underscored unique response patterns among right wing and conservative individuals when confronted with an existential threat to their way of life ( Burke et al., 2013). Drawing on this research, Pyszczynski et al. (2021) suggested that during the COVID-19 pandemic, conservative individuals in the US affirmed their distal defenses by publicly defying federal orders to wear masks in public facilities. Orders which they perceived as an existential threat to their personal freedoms. Along these lines we explored the impact of mortality salience on CCD and whether this impact is unique among conservative individuals in contrast to liberals. We comprised a sample of US residents who defined their political orientation as either Democrats or Republicans. Half of the participants underwent death priming, and the other half pain priming (a control condition). Half of the participants in each condition completed a climate denial questionnaire immediately after their priming (death or pain), while the other completed the questionnaire after a short delay. We hypothesized that in the immediate wake of death priming, CCD would be relatively reduced among all participants (proximal defense condition). However, following death priming and the subsequent delay task, CCD would be relatively heightened among Republican participants (distal defense condition).

## Method

### Participants

The study was approved by the Bar Ilan University Psychology Department ethics committee on November 7th, 2022 (approval number 31\2022). For this preregistered study ( https://osf.io/7ny2g), we recruited 219 participants on Amazon’s MTurk (129 men, M age = 42.52 years, SD = 10.60). Participation was restricted to United States residents who defined their political inclination as either Democrat or Republican (for additional details regarding demographics, see Table 1). To eliminate unreliable data, we removed from the data set entries with 50% and above of missing values. Other flags for eliminating data included clear response set patterns as well as unreasonably short durations of survey completion. While this process may have resulted in the removal of some genuine participants, we erred on the side of caution given the importance of data quality. Considering the sample size and an estimated effect size of 0.25, we had the power of 0.76 as determined by an a priori power analysis (G*Power; Faul et al., 2007, https://www.psychologie.hhu.de/arbeitsgruppen/allgemeine-psychologie-und-arbeitspsychologie/gpower).

**Table 1.** Zero-sum correlations between climate denial and demographic variables.

	CCD	Age	PA	Employment	Income	Education	Gender
CCD							
Age	.10						
PA	-.31 **	.00					
Employment	-.04	-.23 **	-.02				
Income	-.04	.12	.06	-.21 **			
Education	.13	-.12	.13	-.13	.43 **		
Gender	-.03	-.13	-.06	.10	-.15 *	-.04	

Note: CCD-Climate Change Denial, PA-Political Activeness.

* *p*<.05.

** *p*<.01.

### Materials

Mortality salience. We used a 15-item true/false questionnaire to prime thoughts of death ( Templer, 1970). This approach has been utilized successfully in a previous TMT studie conducted via the Amazon’s MTurk ( Boyd et al., 2020). Participants were randomly assigned to answer questions about dying (e.g., “I am very much afraid to die”) or parallel questions about experiencing pain (e.g., “I am very much afraid of being in pain”), which served as the control. As in other research, merely responding to the questions related to death (in contrast to pain) constitutes the mortality salience prime. Therefore, responses to the prompts were not examined.

Climate change denial (CCD). We measured *climate change denial* with an established 16-item scale ( Häkkinen & Akrami, 2014). The scale captures mainly the denial of climate change’s seriousness and the denial of human involvement (e.g., “Warming of the Earth’s climate is natural and does not depend on human influence”). Responses are on a 6-point scale (1 = *do not agree* to 6 = *agree*). After reversing the scale of five items, a total score was computed by averaging the 16 items, with higher scores reflecting higher CCD. In the current sample, Cronbach’s α was 0.85.

### Procedure

After signing the consent form participants completed the priming task. Then they filled the CCD scale immediately after the prime task or after a five-minute delay task consisting of a word puzzle. Following the CCD, participants filled out a demographic questionnaire, probing their age, gender, political orientation and activeness (self-report from zero to seven), education, employment, and income. Overall, the session lasted between 5 and 10 minutes, depending on whether participants completed the delay task. All research conditions were administered and randomized with the Qualtrics software platform ( Qualtrics XM: The Leading Experience Management Software).

## Results

We conducted a three-way ANOVA with prime type (mortality or pain), delay task (with or without) and political orientation (Democrat or Republican) as factors. CCD score was the independent measure.

Results indicated a main effect regarding political orientation, *F* (1,211) *=* 41.45 *, p =* 0. 000 *, η* ^2^ = 0.16; as documented in previous CCD research, in the current sample CCD was significantly higher among Republican participants (M = 3.70; 95% CI [3.53, 3.86], SD = 0.93) in contrast to Democrat participants (M = 2.89; 95% CI [2.70, 3.07], SD = 0.95).

A significant two-way interaction between the prime type and the delay task was also found; *F* (1,211) *=* 4.95 *, p =* 0. 027 *, η* ^2^ = 0.02. According to our hypotheses, we conducted follow-up analysis for each task condition (with or without a delay task corresponding to distal and proximal defenses, respectively). These analyses indicated that the interaction source was from the no delay group, *F* (1, 107) = 4.64, p = 0.033, *η* ^2^ = 0.04). CCD scores in the death condition were lower (M = 3.37; 95% CI [3.16, 3.57], SD = 0.71) than in the pain condition (M = 3.69; 95% CI [3.47, 3.90], SD = 0.85), indicating a reduction in CCD due to death priming in the proximal defense condition.

**Figure 1.**
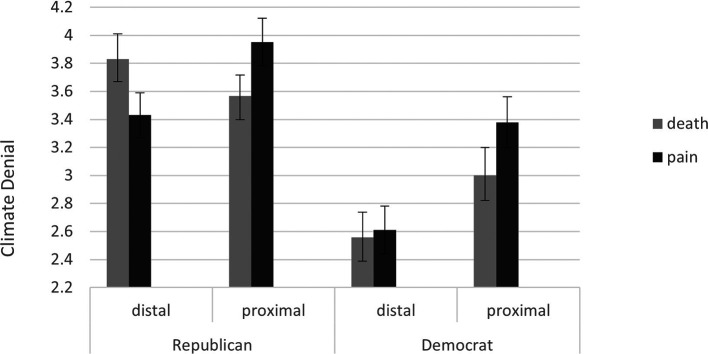
Mean Scores of Climate Change Denial Across the Different Research Groups Among Republican and Democrat Participants. Figure 1 alt text: Among republican participants while in the proximal condition death priming reduced CCD, in the distal condition it increased it.

The three-way interaction was insignificant ( *p* = 0.359). However, as can be seen in Figure 1, following the death prime in the distal condition Republican participants displayed a distinct CCD increase in accordance with our hypothesis. Therefore, we conducted among Republican participants an additional two-way ANOVA with prime type and the delay task as independent variables and CCD as the dependent variable. Only when adding political activeness ( *r* = -.31 with CCD, see Table 1) as a covariate did this analysis achieve significance. *F* (1,109) *=* 9.20 *, p* = 0. 003 *, η* ^2^ = 0.08 *.* In the proximal group, the prime effect, although similar to the overall proximal effect reported above, did not achieve significance; *F* (1, 62) = 2.42, *p* = 0.125). However, the distal effect was significant; *F* (1, 46) = 5.99, *p* = 0.018, *η* ^2^ = 0.115). CCD scores in the death condition were higher ( *M* = 4.01; *95% CI* [3.62, 4.41], *SD* = 0.68) than in the pain condition ( *M* = 3.41; *95% CI* [3.08, 3.73], *SD* = 1.08), indicating an increase in CCD due to death priming within the distal defense mode among Republican participants.

Notably, political activeness (PA) was overall higher among Republican participants ( *M* = 3.86, *SD* = 2.00), in contrast to Democratic participants ( *M* = 3.05, *SD* = 1.90, *t*(208) = 2.97, *p* = 0.003, *d* = 0.41). In turn, undermining the randomness of participant distribution regarding political orientation (see Table 2 for descriptive statistics regarding CCD and political activeness among Republican participants).

**Table 2.** Climate denial scores and political activeness scores of the four republican participant study groups.

Prime Type	Delay Task	Climate Denial	Political Activeness	N
Death	With	3.99 (.68)	3.95 (1.91)	22
	Without	3.57 (.76)	3.18 (2.10)	37
	Overall	3.73 (.76)	3.66 (2.01)	59
Pain	With	3.38 (1.08)	3.59 (1.93)	27
	Without	3.95 (.92)	4.54 (1.99)	28
	Overall	3.67 (1.03)	4.07 (2.00)	55
Overall	With	3.73 (.85)	3.41 (2.00)	49
	Without	3.65 (.97)	4.20 (1.95)	65
	Overall	3.70 (.90)	3.86 (2.00)	114

*Note.* Regarding political activeness in the delay task condition (distal) there were eight missing cases: three in the death prime condition and five in the pain prime condition.

## Discussion

Three main findings were observed in the current study. First, as documented in previous research, beyond the prime type and the delay task variables CCD was higher among Republican participants than Democrat participants. Furthermore, in the immediate wake of mortality priming CCD was reduced among all participants (the proximal defense effect). Finally, following death priming and a delay task, CCD increased among Republican participants when controlling for PA (the distal defense effect).

Indeed, the proximal effect suggests that death priming operates as a tool for CCD reduction when existential threat is in focal attention. Accumulating TMT research has underscored that while individuals attentionally avoid existential threat when it is abstract and does not pose an immediate threat, their focal attention gravitates toward it when perceived to pose an immediate threat ( Hirschberger et al., 2010; Gerber & Anaki, 2018; Gerber et al., 2023). Applying this dynamic to CCD, as the climate crisis transforms from a futuristic abstract threat into an occurring reality, death priming may be an effective tool for facilitating climate action tendencies accordingly. Notably, the CCD scale used in the current study was a rather abstract perception of climate change, as the nature of self-report scales tends to be. Future research should investigate whether the impact of death priming on CCD is more pronounced when the perception of climate change is based on more concrete stimuli such as visceral images.

Finding a preliminary distal effect among Republicans (only when controlling for PA) sheds an important light on the general tendency for CCD among conservative individuals. Seemingly, this tendency, has been considered primarily to reflect conservative individuals’ benefit from maintaining the economic status quo and existing institutional order ( Norgaard, 2019; Clarke et al., 2019). The distal effect finding that the tendency for CCD among Republican participants was facilitated by death priming, supports a differential understanding; Rather than a general anthropocentric attitude of dominance over nature ( Jylhä & Akrami, 2015), CCD reflects conservative individuals need to cope with an existential threat, reflected in the ramifications of climate change, to their worldview and way of life. This line of investigation is warranted and should be pursued in future research to affirm mutual recognition and common ground for promoting climate change mitigation and adaptation.

Before concluding, one must consider the overall limitations of the current study which is preliminary and exploratory in its nature. Notably, this study was conducted among US residents only, reflecting a particular political environment in time and place. Therefore, its convergence with CCD investigations conducted among samples from other world regions is limited. Moreover, although theoretically sound, the distal effect found among Republicans only when controlling for PA should be treated only as a warrant for future, more powered research. Notably, increased PA levels among Republicans in contrast to Democrats may be attributed in part to it’s being measured following mortality salience. Indeed, mortality salience has been shown to arouse PA among conservative individuals as an expression of their distal defenses ( Schindler et al. 2023). Finally, PA’s mean differences patterns among Republicans ( Table 2) as well as its overall covariance with CCD ( Table 1), indicate that PA should be theoretically considered regarding the impact of mortality priming on CCD in future studies. To conclude, the value of the current research is primarily in being conducive to additional research regarding the psychological aspects of CCD. Indeed, the dynamics of coping with existential threats should be an additional perspective in examining CCD tendencies.

### Ethics and consent

The study was approved by the Bar Ilan University Psychology Department ethics committee on November 7th, 2022 (approval number 31\2022).

A written and informed consent was obtained from each participant prior to participation.

## Data Availability

The research data set is publicly available at the OSF depository ( https://osf.io/v9g7s; Gerber, 2024; DOI 10.17605/OSF.IO/V9G7S). This project contains following dataset: OSF _ Climate Change Denial This is in accordance with the terms of the Creative Commons Zero “No rights reserved” data waiver (CC0 1.0 Public domain dedication) ( http://creativecommons.org/publicdomain/zero/1.0/). The research questionnaires and an example of the word puzzle delay task are publicly available at the OSF depository ( https://osf.io/v9g7s). This is in accordance with the terms of the Creative Commons Zero “No rights reserved” data waiver (CC0 1.0 Public domain dedication) ( http://creativecommons.org/publicdomain/zero/1.0/).

## Software availability statement

As mentioned above, all research conditions were administered and randomized with the third party priority software Qualtrics platform ( Qualtrics XM: The Leading Experience Management Software). The outcome data file was analyzed with the third party priority software IBM statistics 22 ( IBM SPSS Statistics).
