# Supplementary material for: The impact of death priming on climate change denial: a preliminary investigation
Source: F1000Res. 2024 Jul 3;13:737. [Version 1] doi: 10.12688/f1000research.151742.1 (PMC11391190; doi:10.12688/f1000research.151742.1)
Supplement: Peer review history

# Reviewer report: Reviewer response for version 1

**DOI:** 10.5256/f1000research.166415.r317847

**Published:** 2024-09-11

**Authors:** Lauren Keira Marie Smith

**Affiliations:** 1 University of Waterloo, Waterloo, Ontario, Canada; 2 Royal Roads University (Ringgold ID: 8202), School of Environment and Sustainability, Victoria, British Columbia, Canada

The authors tackle an important challenge with facing climate change – that of climate deniers. The existential, life-threatening nature of climate change is explored regarding its influence on worldview reinforcement among climate deniers and those with conservative political orientation.

 The authors found that CCD decreases as a proximal mortality defense – a very interesting and necessary finding given the unavoidability of threatening climate reminders in today’s world – and our pressing need to act on climate change. For Republican participants, CCD increased in the distal priming and delay. The role of political activeness and its covariance with CCD is another interesting contribution to the literature. This combination of findings is particularly useful for strategizing around how to communicate existentially threatening issues, like climate change, to those least inclined to believe the threat and perhaps most reluctant to act in pro-environmental ways.

**Recommendations: **Specify US participants in the abstract. It would also be useful to know if participants were required to have a certain MTurk approval rating (to avoid bots and improve reliability, for example) and, if not, to discuss this dynamic in limitations. Including how these findings may influence or can be useful for climate communicators or those hoping to minimize climate change denial would be a useful, practical inclusion.

Is the work clearly and accurately presented and does it cite the current literature?

Yes

If applicable, is the statistical analysis and its interpretation appropriate?

Yes

Are all the source data underlying the results available to ensure full reproducibility?

Yes

Is the study design appropriate and is the work technically sound?

Yes

Are the conclusions drawn adequately supported by the results?

Yes

Are sufficient details of methods and analysis provided to allow replication by others?

Yes

Reviewer Expertise:

Interdisciplinary climate communication; Climate emotions; Pro-environmental behaviour change

I confirm that I have read this submission and believe that I have an appropriate level of expertise to confirm that it is of an acceptable scientific standard.

# Reviewer report: Reviewer response for version 1

**DOI:** 10.5256/f1000research.166415.r314222

**Published:** 2024-08-20

**Authors:** Bablu Kumar Dhar

**Affiliations:** 1 Mahidol University, Salaya, Nakhon Pathom, Thailand

The manuscript makes a valuable contribution to the understanding of how existential threats influence climate change denial, particularly in relation to political orientation. The study’s design is robust, and the results offer intriguing insights that warrant further investigation. However, as a preliminary study, the conclusions should be viewed as tentative, and the need for additional research is clear.

**Recommendations for the Authors:**

1. **Clarify the Generalizability of Findings:** Emphasize the preliminary nature of the findings more explicitly in the conclusion, and discuss the limitations in terms of sample size and generalizability more thoroughly.
2. **Expand on the Implications:** While the discussion of the results is solid, consider expanding on the broader implications of these findings for climate change communication strategies and political psychology.
3. **Consider Additional Analyses:** Given the exploratory nature of the study, additional post-hoc analyses or robustness checks could strengthen the conclusions drawn from the data.

The manuscript is well-executed and provides a strong foundation for future research on the psychological mechanisms underlying climate change denial. With some revisions and further research, the study has the potential to significantly contribute to the field.

Is the work clearly and accurately presented and does it cite the current literature?

Yes

If applicable, is the statistical analysis and its interpretation appropriate?

Yes

Are all the source data underlying the results available to ensure full reproducibility?

Yes

Is the study design appropriate and is the work technically sound?

Yes

Are the conclusions drawn adequately supported by the results?

Partly

Are sufficient details of methods and analysis provided to allow replication by others?

Yes

Reviewer Expertise:

Environmental Management

I confirm that I have read this submission and believe that I have an appropriate level of expertise to confirm that it is of an acceptable scientific standard.
